# Hearth and Home for 1873

**Published:** 1873-02

**Authors:** 


Hearth and Home for 1873.—This illustrated weekly is a treasure in every household. A magnificent new American story, by Edward Eggleston, has already commenced—the general editorial care in its conduct still continues, while instructive and pleasing writers will constantly aid in making the paper a most welcome visitor in every home. As a premium, the publishers have secured a very large and most beautiful painting from which is printed a chromo of eighteen colors to produce the beautiful coloring and shading of the original—which can be secured by all subscribers by remitting three dollars for the *Hearth and Home.* Address, Orange Judd & Co., 245 Broadway, New York.

